# The C2A domain in dysferlin is important for association with MG53 (TRIM72)

**DOI:** 10.1371/5035add8caff4

**Published:** 2012-11-05

**Authors:** Chie Matsuda, Katsuya Miyake, Kimihiko Kameyama, Etsuko Keduka, Hiroshi Takeshima, Toru Imamura, Nobukazu Araki, Ichizo Nishino, Yukiko Hayashi

**Affiliations:** Biomedical Research Institute, National Institute of Advanced Industrial Science and Technology; Department of Neuromuscular Research, National Institute of Neuroscience, National Center of Neurology and Psychiatry; Biomedical Research Institute, National Institute of Advanced Industrial Science and Technology; Department of Neuromuscular Research, National Institute of Neuroscience, National Center of Neurology and Psychiatry; Department of Biological Chemistry, Kyoto University Graduate School of Pharmaceutical Science; Biomedical Research Institute, National Institute of Advanced Industrial Science and Technology; Department of Histology and Cell Biology, School of Medicine, Kagawa University; Department of Neuromuscular Research, National Institute of Neuroscience, National Center of Neurology and Psychiatry; Department of Clinical Development, Translational Medical Center, National Center of Neurology and Psychiatry; Department of Neuromuscular Research, National Institute of Neuroscience, National Center of Neurology and Psychiatry; Department of Clinical Development, Translational Medical Center, National Center of Neurology and Psychiatry

## Abstract

In skeletal muscle, Mitsugumin 53 (MG53), also known as muscle-specific tripartite motif 72, reportedly interacts with dysferlin to regulate membrane repair. To better understand the interactions between dysferlin and MG53, we conducted immunoprecipitation (IP) and pull-down assays. Based on IP assays, the C2A domain in dysferlin associated with MG53. MG53 reportedly exists as a monomer, a homodimer, or an oligomer, depending on the redox state. Based on pull-down assays, wild-type dysferlin associated with MG53 dimers in a Ca2+-dependent manner, but MG53 oligomers associated with both wild-type and C2A-mutant dysferlin in a Ca2+-independent manner. In pull-down assays, a pathogenic missense mutation in the C2A domain (W52R-C2A) inhibited the association between dysferlin and MG53 dimers, but another missense mutation (V67D-C2A) altered the calcium sensitivity of the association between the C2A domain and MG53 dimers. In contrast to the multimers, the MG53 monomers did not interact with wild-type or C2A mutant dysferlin in pull-down assays. These results indicated that the C2A domain in dysferlin is important for the Ca2+-dependent association with MG53 dimers and that dysferlin may associate with MG53 dimers in response to the influx of Ca2+ that occurs during membrane injury. To examine the biological role of the association between dysferlin and MG53, we co-expressed EGFP-dysferlin with RFP-tagged wild-type MG53 or RFP-tagged mutant MG53 (RFP-C242A-MG53) in mouse skeletal muscle, and observed molecular behavior during sarcolemmal repair; it has been reported that the C242A-MG53 mutant forms dimers, but not oligomers. In response to membrane wounding, dysferlin accumulated at the injury site within 1 second; this dysferlin accumulation was followed by the accumulation of wild-type MG53. However, accumulation of RFP-C242A MG53 at the wounded site was impaired relative to that of RFP-wild-type MG53. Co-transfection of RFP-C242A MG53 inhibited the recruitment of dysferlin to the sarcolemmal injury site. We also examined the molecular behavior of GFP-wild-type MG53 during sarcolemmal repair in dysferlin-deficient mice which show progressive muscular dystrophy, and found that GFP-MG53 accumulated at the wound similar to wild-type mice. Our data indicate that the coordination between dysferlin and MG53 plays an important role in efficient sarcolemmal repair.

## Introduction

Dysferlin is a sarcolemmal protein, and dysferlin deficiency causes Miyoshi myopathy (MM) and limb girdle muscular dystrophy type 2B (LGMD2B) [1,2]. Based on the observation that dysferlin accumulates at wound sites in myofibers in a Ca^2+^-dependent manner, dysferlin is thought to mediate Ca^2+^-dependent sarcolemmal repair [3].

Mitsugumin 53 (MG53), also known as muscle-specific tripartite motif 72, is a recently identified protein involved in membrane repair in skeletal muscle [4]. Mice lacking MG53 suffer progressive myopathy [4], similar to dysferlin-null mice [3]. MG53 is localized in intracellular vesicles and plasma membranes in skeletal muscle, and it accumulates at injury sites in an oxidation-dependent, but not Ca^2+^-dependent, manner [4].

MG53 interacts with dysferlin and caveolin-3 to regulate sarcolemmal repair [5]. When expressed in C2C12 myoblasts that lack endogenous MG53, damaged membrane sites cannot be repaired in the presence of GFP-dysferlin, however, co-transfection of MG53 and GFP-dysferlin in these myoblasts results in GFP-dysferlin accumulation at injury sites [5]. These findings indicated that recruitment of dysferlin to the injury site of the membrane depends on MG53. However, it remains unclear whether the absence of dysferlin perturbs recruitment of MG53 to the injury site for membrane repair. A previous report has demonstrated the association of dysferlin with MG53 with co-immunoprecipitation (IP) assays using mouse skeletal muscle and C2C12 myoblasts transfected with dysferlin and MG53 [5]. However, which protein domains participate in this interaction between dysferlin and MG53 and whether this interaction is dependent on Ca^2+^ remain unclear. MG53 oligomerizes via disulfide bonds [4] and forms homodimers via a leucine-zipper motif in the coiled-coil domain [6]. The interaction between dysferlin proteins and MG53 monomers or oligomers has not been characterized in detail. To understand the precise role of dysferlin and MG53 in sarcolemmal repair, it would be helpful to determine whether dysferlin associates with MG53 monomers, oligomers, or both in a Ca^2+^-dependent manner.

Thus, to examine the biological role of the association between dysferlin and MG53, we used the following strategy to examine the effect of the absence of MG53 oligomers on dysferlin. We co-transfected mouse skeletal muscle with wild-type dysferlin-EGFP and RFP-tagged wild-type MG53 or a RFP-tagged MG53 mutant (RFP-C242A –MG53), and conducted a membrane-repair assay using a two-photon laser microscope. The C242A–MG53 mutant has been reported to form dimers, but not oligomers [6]. There is no report of simultaneous observation of dysferlin and MG53 during sarcolemmal repair; however, we have successfully performed real-time imaging of dysferlin-GFP and MG53-RFP after membrane injury in mouse skeletal muscle.

Dysferlin protein is absent or severely reduced in the skeletal muscle of patients with dysferlinopathy [7] and of SJL and A/J mice with mutations in the dysferlin genes [8]. To examine whether the absence of dysferlin affects the recruitment of MG53 to injury sites, we transfected skeletal muscle from dysferlin-deficient SJL and A/J mice with EGFP-MG53 and conducted membrane repair assays. These experiments are helpful in elucidating the molecular pathology of dysferlinopathy and revealed that MG53 accumulated in the skeletal muscles of dysferlin-deficient mice, which develop progressive muscular dystrophy.

We present evidence indicating that efficient sarcolemmal repair requires both dysferlin and MG53.

## Methods

*Immunoprecipitation.* To examine the interaction between MG53 and dysferlin, mouse gastrocnemius muscles were lysed in lysis buffer containing 20 mM Tris-HCl (pH 7.5), 150 mM NaCl, 1% NP-40, and Complete mini EDTA-free protease inhibitor cocktail (Roche) [9] supplemented with 1 mM CaCl_2_ or 2 mM EGTA. Lysates pre-cleared with Protein A/G agarose (Pierce) were incubated with polyclonal antibodies against mouse MG53 [4] or mouse dysferlin; the anti-dysferlin antibody was made in rabbit by injecting bacterial recombinant protein containing residues 1669 to 1790. The immunoprecipitated proteins were separated by SDS-PAGE and detected on immunoblots using the same antibodies used for IP or the anti-human dysferlin monoclonal antibody, NCL-Hamlet (Novocastra Laboratories).

A human MG53 cDNA was amplified by PCR and subcloned into pFLAG-CMV-4 (Sigma). Wild-type and truncated human dysferlin that were each tagged with c-myc were generated previously [10]. We also created five truncated human dysferlin constructs with the C2A domain (aa 1-149, 1-349, and 1-1080) and without the C2A domain (aa 130-2080 and 1081-2080). The sequence of each construct was verified by DNA sequencing. FuGENE 6 or E-xtremeGENE 9 (Roche) was used to transiently transfect COS-7 cells with MG53 and wild-type or mutant dysferlin constructs. Transfectants were cultured for 48 h and subsequently lysed in the same lysis buffer used to lyse mouse muscle, except that this buffer lacked CaCl_2_ and EGTA. Lysates pre-cleared with Protein G-Sepharose (GE Healthcare) were incubated with anti-FLAG (M2, Sigma) or anti-c-myc (9E10, Santa Cruz Biotechnology) monoclonal antibodies; Protein G-Sepharose was then added. Immunoprecipitated proteins were analyzed by immunoblotting using M2 and anti-c-myc polyclonal (A14, Santa Cruz Biotechnology) antibodies.

*Pull-down assay. *Fragments of the dysferlin C2A domain (corresponding to aa 1-129 of human dysferlin) were amplified as cDNA by PCR and subcloned into pGEX-5X-3 (GE Healthcare). Dysferlin p.W52R (TGG to CGG at c.527-529) and p.V67D (GTG to GAT at c.572-574) mutations were introduced by PCR using appropriate primers. GST fusion proteins expressed in BL21 *E. coli* were purified using sarkosyl [11] and bound to glutathione Sepharose 4B (GE Healthcare). COS-7 cells overexpressing FLAG-tagged human MG53 were lysed in lysis buffer containing 10 mM Na_2_HPO_4_, 1.8 mM KH_2_HPO_4_, 1% NP-40 (pH 7.4), 2 mM EGTA, various concentration of CaCl_2_, and Complete mini EDTA-free protease inhibitor cocktail. EGTA was used to chelate the free Ca^2+^ in solution and CaCl_2 _at various concentrations. The free calcium concentration was calculated using the free software CALCON3.6. Lysates were centrifuged to remove cellular debris, supplemented with 5 mM N-methylmaleimide (NEM) or 5 mM dithiothreitol (DTT), and finally subjected to protein cross-linking by treating with 2 mM glutaraldehyde (GA) for 5 min at room temperature, which was quenched with 100 mM Tris-HCl (pH 7.5) [6]. The cross-linked lysates were diluted with 75 mM Tris-HCl (pH 7.5), 150 mM NaCl, 1% NP-40, 2 mM EGTA, various concentrations of CaCl_2_, and Complete mini EDTA-free protease inhibitor cocktail. Lysates pre-cleared with GST bound to glutathione Sepharose 4B were divided into aliquots and incubated with wild-type, p.W52R, and p.V67D dysferlin C2A-GST fusion protein bound to beads for 2 hr at 4°C. After three washes in lysis buffer containing 75 mM Tris-HCl (pH 7.5), 2× sample buffer (125 mM Tris-HCl (pH 6.8), 4% SDS, 20% (v/v) glycerol, and 0.004% bromophenol blue) was added to the beads, and the mixtures were incubated for 10 min at 85°C. Bound proteins were separated by SDS-PAGE and subjected to immunoblotting with the anti-FLAG antibody M2.

*In vivo transfection and membrane repair assay.* Twenty micrograms of N-terminal RFP-tagged human MG53 cDNA/pcDNA3.1 and/or C-terminal GFP-tagged human dysferlin cDNA/pcDNA3.1 plasmid DNA were injected into the flexor digitorum brevis of anesthetized, 4-week-old male C57BL6J and dysferlin-deficient SJL and A/J mice. Electroporation of plasmid DNA was performed using an electric pulse generator (CUY21SC, NEPAGENE) as described previously [12]. Seven days after electroporation, skeletal muscle myocytes (for whole-mount viewing) or individual myofibers were isolated and subjected to plasma membrane injury created by a two-photon laser microscope, LSM 710NLO with GaAsp Detectors (Zeiss) and Chameleon Vision II System (Coherent)[3]. Myofiber wounding using the 820-nm infrared laser and resealing analysis based on the kinetics and extent of FM1-43 or 4-46 dye (Molecular Probes) entry through open disruptions was carried out as previously described [3,13,14].

*Ethics Statement. *All experiments involving animals were performed according to the Procedure for Handling Experiments involving Animals of AIST (National Institute of Advanced Industrial Science and Technology) and approved by the Institutional Animal Care and Use Committee of AIST.

## Results

**Association of MG53 and dysferlin in mouse skeletal muscle**

We used an IP assay with protein from mouse muscle to confirm that endogenous MG53 associates with dysferlin *in vivo*. MG53 and dysferlin associated only in the absence of EGTA and CaCl_2_ (Fig. 1). The same result was obtained using C2C12 myotubes (data not shown). MG53 was specifically co-immunoprecipitated by the anti-dysferlin antibody, and conversely dysferlin was specifically co-immunoprecipitated by the anti-MG53 antibody. Thus, we confirmed that endogenous MG53 and endogenous dysferlin form a protein complex in mouse skeletal muscle without EGTA or CaCl_2_ supplementation.

**IP assay of dysferlin and MG53.**
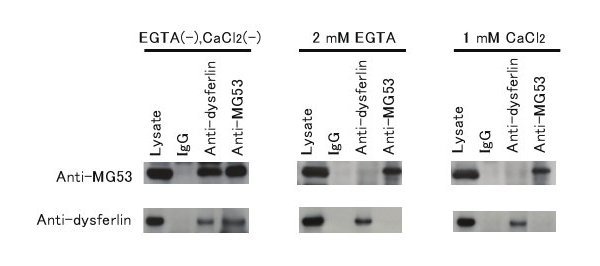
MG53interacts with dysferlin in mouse skeletal muscle. Extracts from wild-type mouse skeletal muscle were subjected to IP with polyclonal anti-MG53 antibodies or polyclonal anti-dysferlin antibodies. Immunoprecipitated proteins were subjected to SDS-PAGE and visualized on immunoblots treated with the same antibodies that were used for IP.

**Identification of the MG53-associating domain of dysferlin**

Next, we used IP to define the region of dysferlin that associates with MG53. Specifically, we used transient co-transfection to introduce a construct encoding full-length human MG53 tagged with FLAG and a construct encoding human dysferlin tagged with c-myc into COS-7 cells; for each co-transfection, full-length dysferlin or one of five deletion mutant forms of tagged dysferlin was used (Fig. 2). For deletion mutants that lacked the C-terminal domain of dysferlin, the transmembrane domain of dysferlin was retained to increase protein stability [10]. Transfectants were lysed in the same buffer that was used for IP assays of mouse skeletal muscle extract, except that this buffer lacked EGTA and CaCl_2_. Full-length dysferlin and deletion mutants that retained the N-terminal C2 (C2A) domain of dysferlin were co-immunoprecipitated by anti-MG53 antibody. In contrast, dysferlin mutants that lacked this N-terminal domain, Δ2-1080 and Δ2-129, failed to interact with MG53. These results indicated that the C2A domain of dysferlin was necessary for association with MG53.

**Identification of MG53-binding region of dysferlin.**
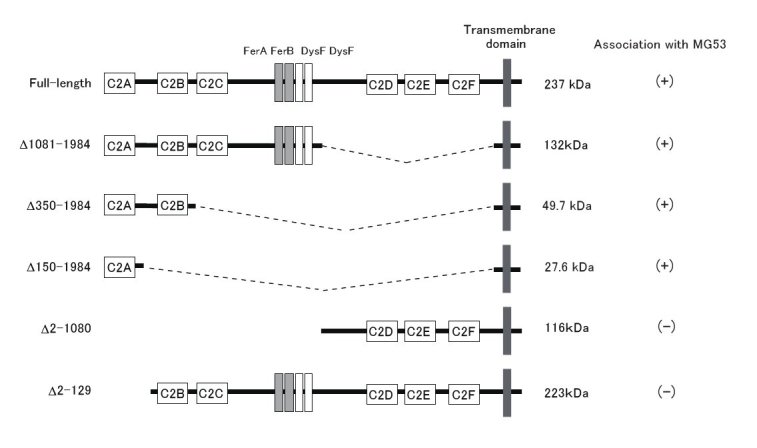
The dysferlin C2A domain associates with MG53. Constructs encoding dysferlin deletion mutants were used for co-IP assays, and the results of these experiments are shown on the right. Deletion mutants encoding c-myc-tagged dysferlin mutants and FLAG-tagged full-length MG53 were co-expressed in COS-7 cells. IP and immunoblotting were performed using antibodies against the c-myc and FLAG tags. MG53 was co-immunoprecipitated with full-length dysferlin and the dysferlin mutants that lacked the C-terminus, but not with the dysferlin mutants that lacked the N-terminus.

**Characterization of the association of dysferlin C2A domain with MG53 monomers and MG53 oligomer**

C2 domains are known to bind to phospholipids and/or proteins in a Ca^2+^-dependent or Ca^2+^-independent manner [15]. Therefore, we used a pull-down assay to examine whether the Ca^2+^ concentration affected the association between MG53 and the dysferlin C2A domain. We used lysis buffer containing 75 mM Tris to reduce the change in pH that can result from the addition of CaCl_2_, to examine the calcium-dependency of the association between dysferlin and MG53. Reportedly, MG53 can exist as a monomer or an oligomer, depending on the redox state [4]. We used DTT for monomerization of MG53 by reducing sulfhydryl groups. Addition of 5 mM DTT resulted in complete dissociation of all MG53 oligomers (Fig. 3). To conduct a pull-down assay for MG53 oligomers, we treated cell lysates with an alkylating reagent, NEM, which reacts with sulfhydryl groups to form stable thioether bonds [6]. Multimers of MG53 were stabilized by chemical cross-linking with GA. Addition of 5 mM NEM to cell lysates resulted in oligomerization of MG53 (Fig. 3). In the presence or absence of Ca^2+^, MG53 oligomers associated with wild-type C2A-GST, whereas MG53 monomers did not associate with wild-type C2A-GST. In the absence of DTT or NEM, MG53 existed as oligomers including dimers, which associated with WT C2A-GST only in 10 mM free Ca^2+ ^(Fig. 3, top).

Next, we generated two mutant versions of C2A-GST (W52R or V67D) to further characterize the association between MG53 and the C2A domain. A V67D missense mutation in the human dysferlin gene has been found in patients with MM and patients with LGMD2B [16]; similarly, the W52R dysferlin missense mutation has been found in patients with LGMD2B [17]. Each mutant C2A-GST, like the wild-type C2A, associated with MG53 oligomers when conditions included NEM in the presence or absence of Ca^2+^ (Fig. 3). However, the V67D mutation altered the calcium sensitivity of the association between C2A-GST and MG53 dimers; specifically, V67D-C2A-GST could associate with MG53 when conditions did not include NEM in the absence of Ca^2+^. In contrast, W52R-C2A-GST did not associate with MG53 when conditions did not include NEM in the presence or absence of Ca^2+^. These results revealed that the V67D mutation in the dysferlin C2A domain altered the Ca^2+^-dependence of the association between dysferlin and MG53 dimers.

**Pull-down assay of dysferlin C2A-GST and MG53.**
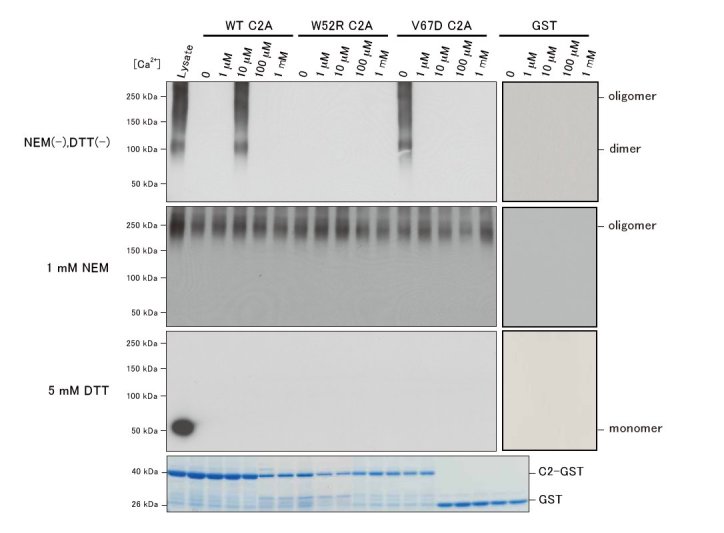
COS-7 cells overexpressing FLAG-tagged MG53 were lysed and supplemented with DTT or NEM, and proteins in these lysates were cross-linked with GA. Cross-linked proteins were incubated with glutathione Sepharose 4B beads bound to wild-type C2A-GST, V67D C2A-GST, or GST. GST fusion proteins bound to beads were separated by SDS-PAGE, followed by Coomassie Brilliant Blue R-250-staining. Precipitated MG53 oligomers/monomers were detected on immunoblots using an anti-FLAG antibody. Mutations in the C2A domain affect the association of between dysferlin and MG53.

**MG53 with a C242A missense mutation shows impaired accumulation at wound sites and attenuates the formation of dysferlin patches**

To examine the biological role of the association between dysferlin and MG53 in sarcolemmal repair, we used mouse skeletal muscle co-transfected with dysferlin-EGFP and RFP-tagged wild-type MG53 or RFP-tagged mutant MG53 to perform a membrane repair assay. The mutant MG53 carried a C242A missense mutation and is designated RFP-C242A-MG53 here. MG53 with a C242A missense mutation reportedly exists as a monomer or dimer when expressed in mammalian cells, but does not form oligomers via disulfide bonding [4,6]. RFP-C242A-MG53 did not accumulate at wound sites as reported previously, and it was associated with defective sarcolemmal repair [4]. Co-expression of RFP-C242A-MG53 did not affect the subcellular localization of dysferlin in myofibers, and dysferlin was localized in a striated pattern (Fig. 4A). However, RFP-C242A-MG53 compromised the accumulation of dysferlin at injury sites (Fig. 4A, B). When the movement of dysferlin and wild-type MG53 were observed simultaneously in mouse skeletal muscle, RFP-wild-type MG53 accumulated more slowly at injury sites than dysferlin-EGFP (Fig. 4A). Accumulation of dysferlin-EGFP at wound sites stops within 5 seconds of injury and disperses gradually, while wild-type MG53 continues to accumulate for 200 seconds after injury (Fig. 4A and 4B).

**Figure:**
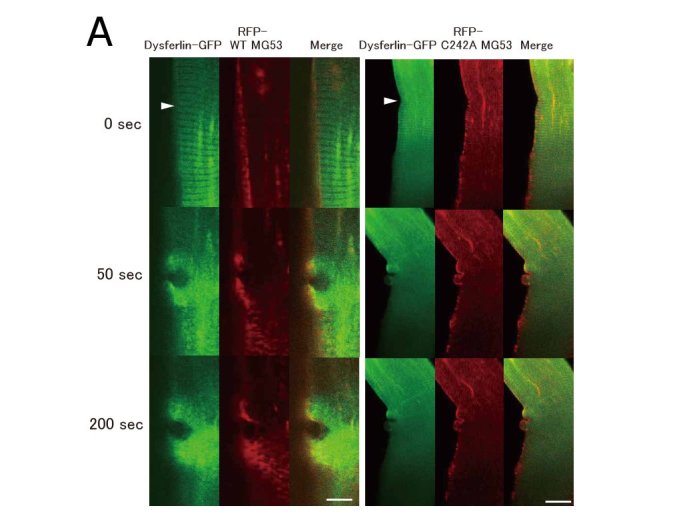

**Membrane repair assay of myofiber transfected with dysferlin-GFP and RFP-MG53.**
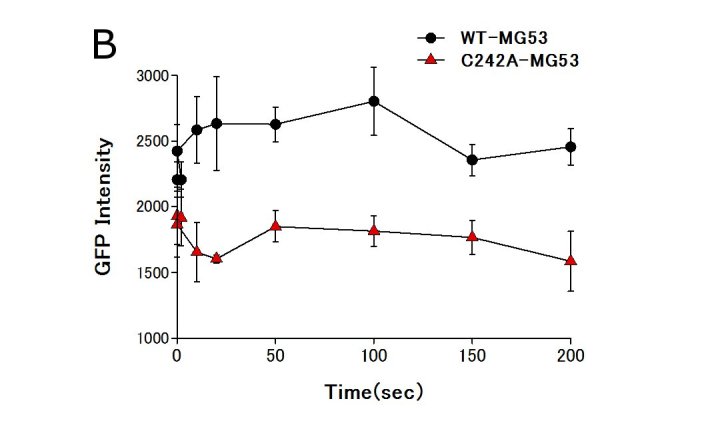
RFP-C242A MG53 perturbed the accumulation of dysferlin at wound sites in the sarcolemma. A. Dysferlin-GFP was simultaneously expressed with RFP-tagged wild-type MG53 or the RFP-C242A-MG53 mutant in mouse skeletal muscle. Arrowheads indicate sites of membrane injury, which were induced with a two-photon laser microscope. Dysferlin-GFP accumulated at the injury site in the presence of RFP-wild-type MG53, but no obvious accumulation of dysferlin-GFP was observed in the presence of the RFP-C242A-MG53 mutant. Scale bar, 10 mm. B.Time course fluorescence intensity (n=3) at wounded sites versus time. For every image taken, the fluorescence intensity of dysferlin-GFP at the site of the damage (circle of 5 mm in diameter) was measured with Zeiss LSM5 Image Examinar software. Data are means ± standard deviation.

**MG53 accumulates normally at injury site of sarcolemma in dysferlin-deficient mice.**

A previous study revealed that exogenous expression of MG53 in undifferentiated C2C12 cells was necessary for recruitment of GFP-dysferlin to sites of injury [5]. Conversely, to examine whether the recruitment of MG53 requires dysferlin, and to elucidate the molecular pathology of dysferlinopathy, we used skeletal muscle from dysferlin-deficient A/J mice transfected with EGFP-MG53 to perform a membrane repair assay. We confirmed that EGFP-MG53 accumulated at sites of injury (Fig. 5). Sarcolemmal repair was observed and confirmed by FM4-46-loading in A/J mice (data not shown). The accumulation of MG53 at the sarcolemmal wound was observed in A/L mice, similar to wild-type mice. Similar results were obtained from the membrane repair assay using dysferlin-deficient SJL mice.

**Membrane repair assay of myofiber using dysferlin-deficient myofiber transfected with GFP-MG53.**
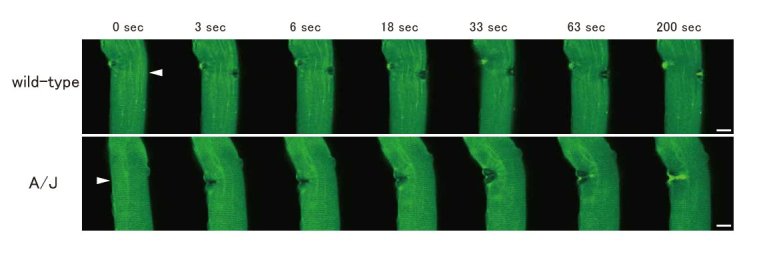
GFP-MG53 accumulated at sites of injury in the sarcolemma in dysferlin-deficient A/J mice, similar to wild-type mice. GFP-MG53 was expressed in wild-type or dysferlin-deficient A/J mice, and a membrane repair assay was performed using transfected myofibers. Subcellular localization of GFP-MG53 was similar between wild-type and A/J mice. Arrowheads indicate membrane injury sites, which were induced with a two-photon laser microscope. Scale bar, 10 μm.

## Discussion

Both dysferlin and MG53 are involved in membrane repair after injury in skeletal muscle. Dysferlin accumulates at wounded sarcolemmal sites, and this accumulation requires the influx of Ca^2+^ into the myofiber [3]. MG53 forms oligomers at the sarcolemmal injury site in an oxidation-dependent manner [4,6]. MG53 associates with dysferlin and facilitates vesicle trafficking to the site of membrane injury, and a recent finding suggests that MG53 and dysferlin may form a complex that participates in membrane repair in striated muscle [5]. To characterize the association between dysferlin and MG53, we used an IP assay and mouse muscle extract with or without exogenously added EGTA or CaCl_2_ to examine the Ca^2+^ dependency of this association. Using lysis buffer that lacked EGTA and CaCl_2_, we observed the association of dysferlin with MG53 in mouse skeletal muscle. Lysates lacking exogenously added EGTA and CaCl_2_ contain physiological concentrations of free calcium. Hence, low concentrations of calcium are likely to be necessary for the interaction between MG53 and dysferlin.

Our results indicated that MG53 oligomers associated with the dysferlin C2A domain in the presence or absence of Ca^2+^, whereas MG53 dimers associated with the dysferlin C2A domain in a Ca^2+^-dependent manner. We also revealed that pathogenic mutations in the dysferlin C2A domain (W52R and V67D) alter the association between this domain and MG53 dimers in a pull-down assay. In the absence of EGTA or Ca^2+^, dysferlin with a C2A missense mutation (W52R or V67D) did not associate with MG53 in an IP assay that used extracts from co-transfected COS-7 cells; however, full-length dysferlin with the most common pathogenic mutation found in Japan, a W999C missense mutation in the dysferlin domain, did associate with MG53 in these IP assays (data not shown). These results indicate that the dysferlin C2A domain is important for the association between dysferlin and MG53. Amino acid W52 in human dysferlin is located between the b5-sheet and the b6-sheet, and V67 is located in the b6-sheet [18]. Both residues are reportedly important for the C2 structure, particularly those of the b-sheet, and are predicted to coordinate calcium [18].

Recently, MG53 was reported to form homodimers, which are essential for MG53-mediated sarcolemmal repair [6]. We used pull-down assays to investigate associations between MG53 monomers or MG53 dimers and the dysferlin C2A domain, and we found that MG53 dimers associated with dysferlin in a Ca^2+^-dependent manner. An increase in the cytoplasmic Ca^2+^ level is necessary for dysferlin accumulation at wounded sarcolemmal sites [3]. The intracellular Ca^2+^ level is maintained at 50-100 nM in resting mammalian cells, but this increases to 6 μM after membrane puncture in Swiss-3T3 cells [19]. The influx of extracellular Ca^2+^ through the wound site is required for vesicle fusion with the plasma membrane and formation of a repair patch in skeletal muscle, but MG53 trafficking to the wound site does not require Ca^2+^ [4]. In pull-down assays in the present study, we demonstrated a selective association between the wild-type dysferlin C2A domain and MG53 dimers at a free Ca^2+^ concentration of 10 μM, but not at lower or higher free Ca^2+^ concentrations. These findings indicated that the concentration of free Ca^2+^ is important for association of dysferlin with MG53 dimers, and suggest that MG53 dimers not only form oligomers, but also associate with dysferlin in response to sarcolemmal injury. The altered Ca^2+^ sensitivity of the association between dysferlin with a mutation in the C2A domain and MG53 dimers in the pull-down assay also suggested that the C2A domain was important in the Ca^2+^-dependent association between dysferlin and MG53 dimers.

We were able to analyze the movement of dysferlin and MG53 in real time during sarcolemmal repair in a membrane repair assay that employs mouse myofibers that express dysferlin-EGFP and RFP-MG53. This is the first report to demonstrate that dysferlin and MG53 have different accumulation patterns at wound sites, and this result indicated that dysferlin and MG53 have different functions in sarcolemmal repair. Our studies also revealed that MG53 carrying a C242A missense mutation can suppress the accumulation of dysferlin at the wound site; this finding, together with results from pull-down assays, suggests that MG53 dimers play an important role in sarcolemmal repair.

Our studies also revealed that MG53 accumulated at injury sites in the sarcolemma in dysferlin-deficient mice, similar to wild-type mice. However, dysferlin-deficient SJL and A/J mice have a progressive muscular dystrophy phenotype, suggesting that MG53 is necessary but not sufficient for efficient sarcolemmal repair.

## Competing Interests

The authors have declared that no competing interests exist.

## Correspondence

Address for correspondence : c-matsuda@aist.go.jp (C. Matsuda)
